# Impending rupture of abdominal aortic aneurysm due to spontaneous obstruction of aortocaval fistula after endovascular abdominal aortic aneurysm repair

**DOI:** 10.1016/j.jvscit.2021.02.002

**Published:** 2021-02-26

**Authors:** Tsuyoshi Fujimiya, Yuki Seto, Keiichi Ishida, Shinya Takase, Hirono Satokawa, Hitoshi Yokoyama

**Affiliations:** Department of Cardiovascular Surgery, Fukushima Medical University, Fukushima, Japan

**Keywords:** Abdominal aortic aneurysm, Aortocaval fistula, Endovascular aortic aneurysm repair

## Abstract

Endovascular aortic aneurysm repair (EVAR) is a valid treatment for patients with abdominal aortic aneurysm with aortocaval fistula. However, an endoleak can be caused by persistent communication between the aneurysm and the inferior vena cava. We present a case of impending rupture due to spontaneous obstruction of an aortocaval fistula after EVAR. Spontaneous obstruction of an aortocaval fistula is rare; however, when occurs, it will cause an endoleak, followed by dilatation or impending rupture of the abdominal aortic aneurysm. EVAR alone for aortocaval fistula will sometimes not be adequate if the type II endoleak is patent.

Endovascular aortic aneurysm repair (EVAR) for abdominal aortic aneurysm (AAA) with aortocaval fistula (ACF) is a valid treatment. However, postoperative endoleak occurs is frequently associated with persistent communication between the aneurysm and the inferior vena cava. It remains controversial whether a persistent fistula after EVAR should be treated. A recent report showed that persistent ACF after EVAR can be managed conservatively if no right heart overload or aneurysm enlargement is present.^1^ We report a case of an AAA, in which rapid dilatation of the aneurysm sac associated with spontaneous obstruction of the ACF was observed after EVAR. The patient provided written informed consent for the report of his case.

## Case report

**Fig 1:**
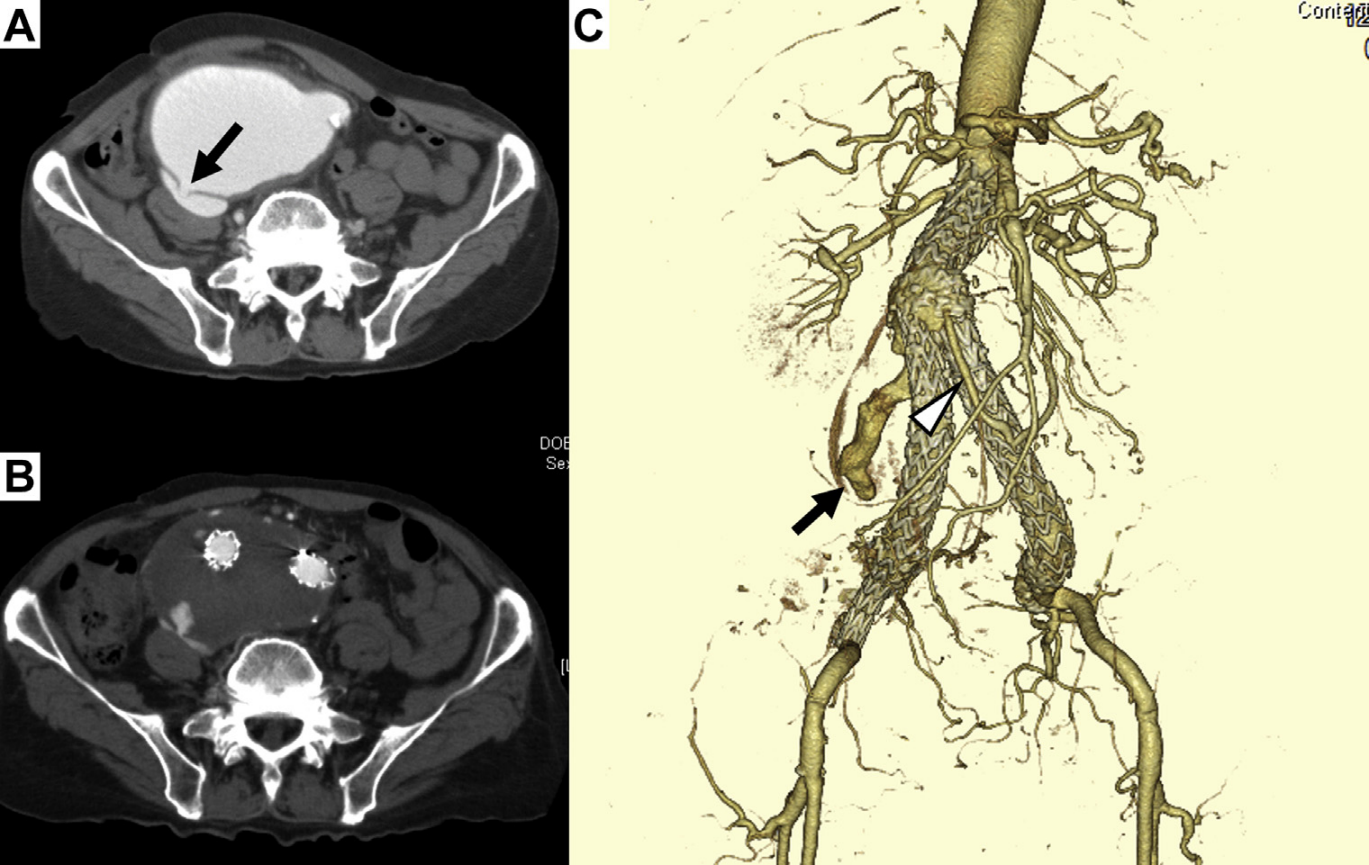
Computed tomography (CT) angiogram at the first operation. **A,** Preoperative CT scan showing abdominal aortic aneurysm (AAA) and aortocaval fistula (ACF; *arrow*). **B,** Postoperative CT scan showing a type II endoleak and persistent communication between the AAA and inferior vena cava (*IVC*); however, the aneurysm sac had shrunk. **C,** Three-dimensional CT angiogram showing persistent communication (*arrow*) via a meandering mesenteric artery (*open arrow*).

An 85-year-old man with a history of smoking, hypertension, and chronic obstructive pulmonary disease had presented with acute abdominal pain and was transferred to the emergency room of our hospital. He had hepatorenal failure and right-sided heart failure. Laboratory testing showed an aspartate transaminase of 1058 U/L, alanine transaminase of 669 U/L, creatinine concentration of 2.77 mg/dL, and brain natriuretic peptide of 1651 pg/mL. Echocardiography revealed distention of the right ventricle and elevation of the tricuspid regurgitation pressure gradient (33 mm Hg). He had undergone emergency EVAR using a bifurcated Endurant II (Medtronic Inc, Santa Rosa, Calif) for an AAA with ACF 4 months previously. Echocardiography 3 days after primary surgery had shown improvement of the right ventricular dilatation and tricuspid regurgitation pressure gradient (22 mm Hg). Tricuspid regurgitation was not observed. At 2 months after EVAR, computed tomography (CT) showed a type II endoleak from the inferior mesenteric artery (IMA) and persistent communication between the aneurysm and inferior vena cava. He was discharged 50 days after emergency EVAR because of shrinkage of the aneurysm sac (the maximum transverse diameter had decreased from 78 to 61 mm; Fig 1) and improvement of the right heart overload.

**Fig 2:**
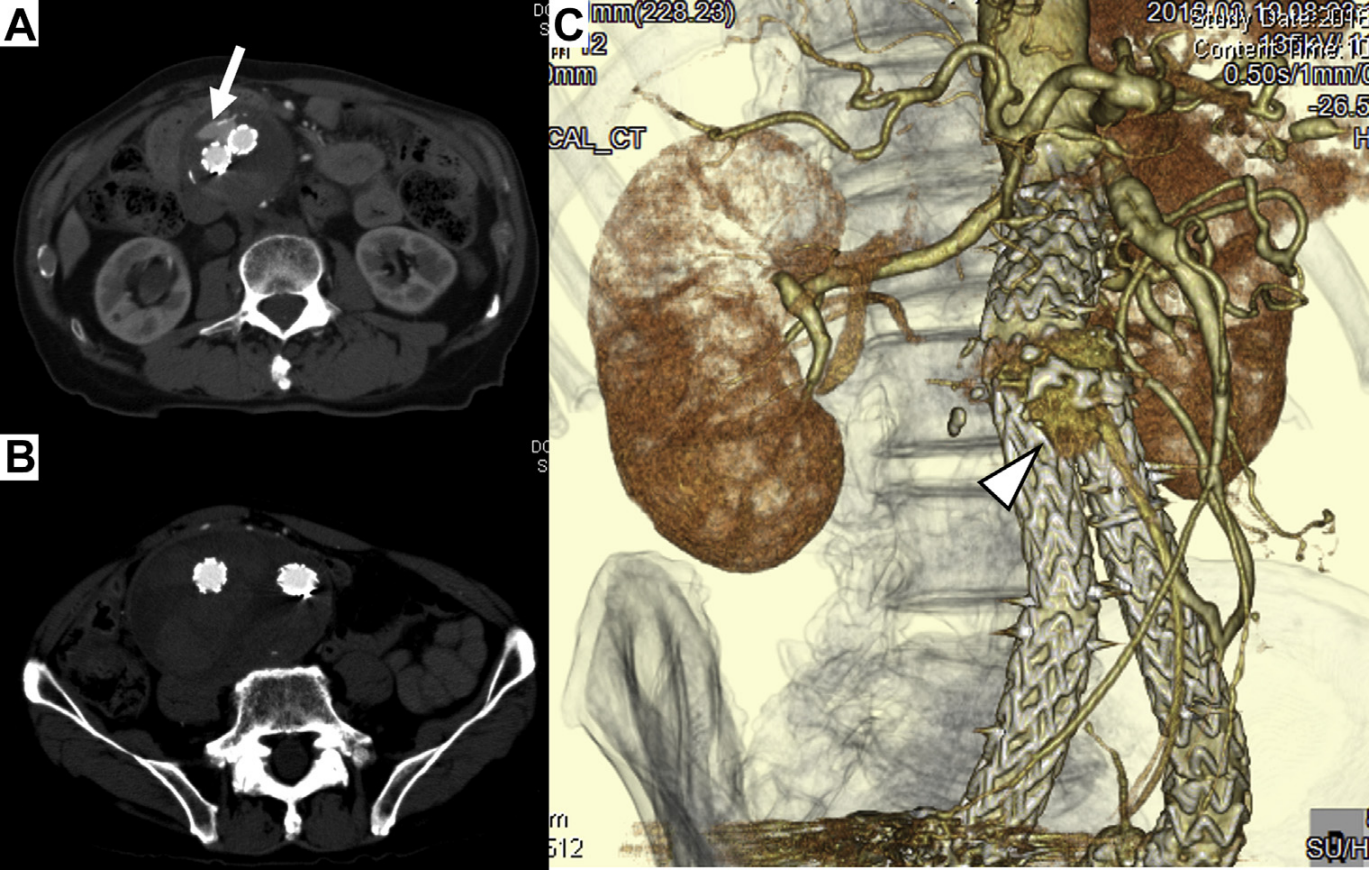
Computed tomography (CT) angiogram at the present admission (4 months after the first operation). CT scan revealing type II endoleak (*arrow*; **A**), rapid dilatation of the aneurysm sac (maximum transverse diameter had increased by 15 mm after first operation) and spontaneous obstruction of the aortocaval fistula (ACF; **B**). **C,** Three-dimensional CT angiogram showing disappearance of ACF (*open arrowhead* indicates type II endoleak via a meandering mesenteric artery).

**Fig 3:**
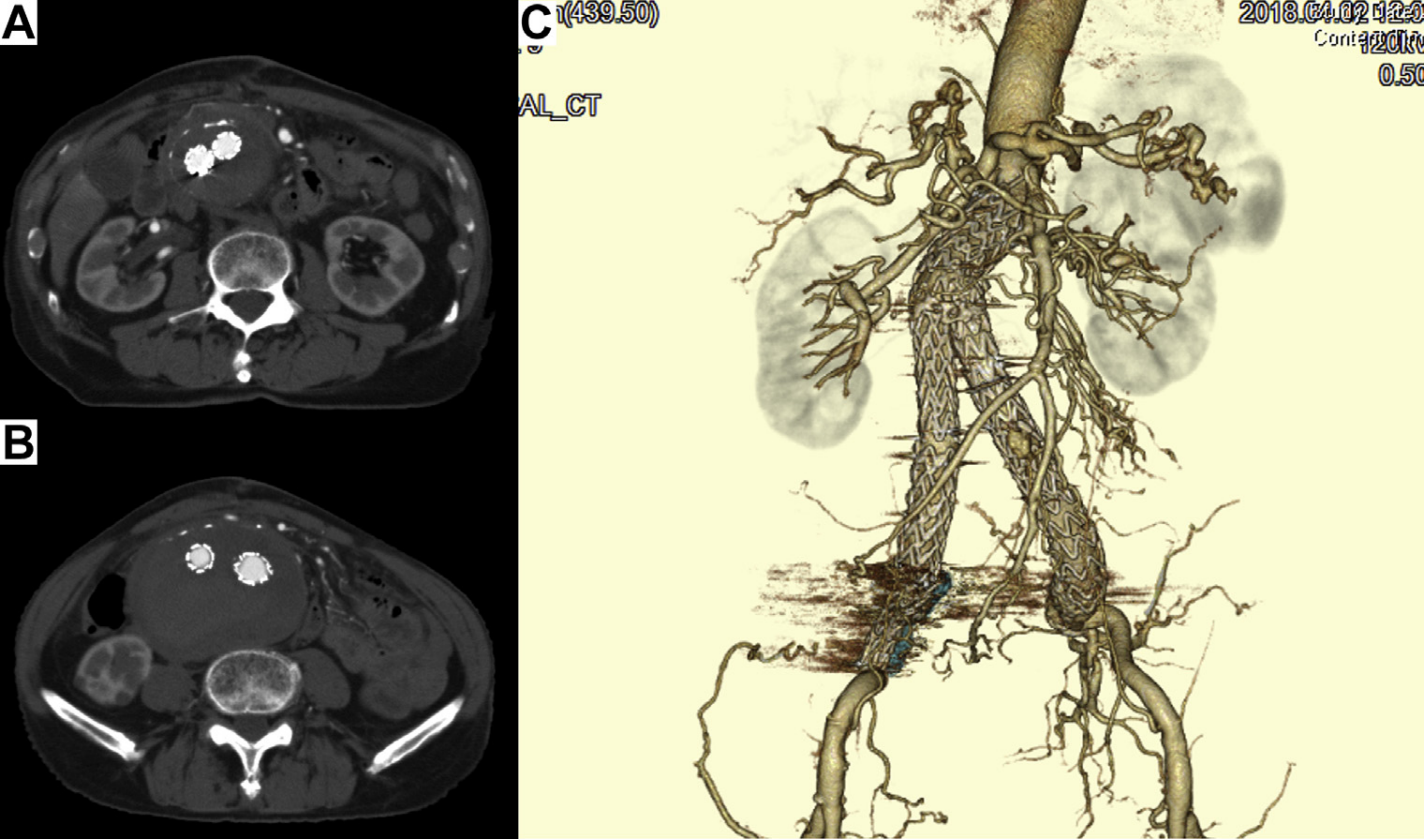
Postoperative computed tomography angiogram showing disappearance of type II endoleak **(A)** and shrinkage of aneurysm sac (maximum transverse diameter decreased by 9 mm; **B**). **C,** Three-dimensional CT scan showing disappearance of type II endoleak.

On admission, his blood pressure was 125/68 mm Hg and his heart rate was 88 beats/min. The physical examination revealed abdominal tenderness and nonpulsatile abdominal mass. No clinical signs of right heart failure or venous hypertension were observed. The results of the laboratory tests were unremarkable. However, CT recorded in the arterial and delayed phases revealed rapid dilatation of the aneurysm sac during the previous 2 months (maximum transverse diameter had increased from 61 to 76 mm) and manifestation of the type II endoleak via the IMA from the meandering mesenteric artery (“dead-end” endoleak) by spontaneous obstruction of the ACF (Fig 2). We performed direct ligation of the IMA with a transperitoneal approach under median laparotomy. Completion angiography did not show any endoleak. The 1-week postoperative CT scan showed shrinkage of the aneurysm sac (the maximum transverse diameter had decreased from 76 to 67 mm) and disappearance of the type II endoleak (Fig 3). Although we performed CT angiography in the arterial phase and delayed phase, we were not able to detect any endoleak. The patient had an uneventful recovery and was discharged 20 days after surgery.

## Discussion

We encountered a case of impending AAA rupture due to spontaneous occlusion of an ACF after EVAR. Spontaneous occlusion of an ACF is rare; however, when it occurs, it can cause a “dead-end” endoleak, followed by dilatation or impending rupture of the AAA.

We found two important clinical issues. First, spontaneous obstruction of the ACF might occur in the remote period after EVAR. Persistent ACF after EVAR has been reported; however, the natural history of the fistula remains unknown. No randomized control trial has been performed to assess treatment of ACF. It remains controversial whether we should treat persistent ACF. Two case studies have reported the usefulness of conservative management for persistent ACF after EVAR.^1^^,^^2^ In one case, the aneurysm sac had shrunk, without disappearance of the persistent ACF at 1 year after EVAR. In the other case, the type II endoleak had disappeared at 4 weeks after EVAR, with shrinkage of the aneurysm sac 1 year later. Neither patient experienced vascular-related events. We chose conservative management for our patient, because no systemic manifestations of right heart overload and venous hypertension were present. To the best of our knowledge, no studies have reported on vascular-related events that occurred because of persistent ACF after EVAR. In the present case, spontaneous obstruction of the ACF had occurred at 4 months after EVAR to treat the AAA. Such obstruction can lead to the manifestation of an endoleak (“dead-end” endoleak). Consequently, rapid dilatation of the aneurysm sac resulted in a state of impending rupture.

Second, EVAR alone without intervention for ACF could be inadequate. We selected conservative strategy for our patient because of previous studies that had reported the usefulness of conservative management. Antoniou et al^3^ found no evidence to sustain the expectation that the AVF might persist in the presence of an endoleak after EVAR for ACF. Simultaneously, they reported that in a few cases of type II endoleak that had remained after EVAR and were observed conservatively, the fistula and type II endoleak had resolved spontaneously.^3^ In contrast, several studies have reported on the usefulness of intervention for ACF rather than conservative management.^4^ In previous reports, the rate of an endoleak after EVAR for ACF was ~50%. More than one half of them had not resolved spontaneously, and these cases had remained aortocaval patent. Persistent ACF has traditionally been treated with direct closure after open repair. In previous reports, an Amplatzer duct occluder and Amplatzer ventricular septal defect occluder were used for the treatment of a persistent ACF.^5^^,^^6^ Bernstein and Jimenez^7^ introduced the kissing technique for a thrombosed inferior vena cava after EVAR for persistent ACF. The presence of an endoleak in association with an ACF can cause inopportune results, including worsening heart failure, disseminated intravascular coagulation, edema of the lower extremities, and continued aneurysm sac enlargement. Moreover, some surgeons have claimed that endoleak and ACF management should be aggressively performed. However, these therapies were off-label use; thus, we must judge the indication of endovascular intervention for arteriovenous communication. However, in the present case, performance of emergent reintervention was required although the ACF was not the direct cause of the manifestation of the type II endoleak. The situation of the aneurysm sac could have changed owing to intervention to treat the ACF after the first operation. A few studies have reported disadvantages of conservative strategy for ACF, although some studies have reported good results with conservative management. We believe that EVAR alone will sometimes be inadequate as a treatment of ACF. The criteria for intervention to treat ACFs are not clear and have remained controversial; thus, further research is required in the future.

## Conclusions

In the present case, spontaneous obstruction of the ACF in the remote period resulted in the development of a “dead-end” (type II) endoleak. We believe that the presence of the “dead-end” endoleak resulted in rapid aneurysm sac expansion.

We recognize that EVAR alone for ACF will be insufficient if an IMA or large lumbar arteries are present. Treatment of a persistent fistula can be performed if the fistula is present in the postoperative phase.
